# Challenging Diagnosis in NUT Carcinoma

**DOI:** 10.1177/10668969211019532

**Published:** 2021-06-09

**Authors:** Grosse Claudia, Grosse Alexandra

**Affiliations:** 1 Institute of Pathology, Kepler University Hospital, Linz, Austria; 2 Institute of Pathology and Molecular Pathology, 27243 University Hospital Zurich, Zurich, Switzerland

**Keywords:** nuclear protein in testis, fluorescence in-situ hybridization, next-generation sequencing

## Abstract

Nuclear protein in testis (NUT) carcinoma represents a highly aggressive, poorly differentiated carcinoma that is genetically defined by rearrangement of NUT gene. The histomorphological appearance ranges from entirely undifferentiated carcinoma to carcinoma with prominent squamous differentiation. NUT carcinoma can display neuroendocrine features. Although it is typically distributed along the midline axis, it may manifest in nonmidline locations. The majority of patients develop rapidly disseminated disease. We illustrate 2 cases of NUT carcinoma, one located in the lung, which closely resembled a neuroendocrine carcinoma, and the other one with assumed lung origin demonstrating metastatic dissemination with diffuse bone involvement, which was clinically first suspected to be a hematological malignancy. Due to its undifferentiated nature, NUT carcinoma may be confused with many entities. NUT immunohistochemistry is considered to be sufficient for the diagnosis. Fluorescence in-situ hybridization analysis and next-generation sequencing are currently used to confirm the diagnosis.

Nuclear protein in testis (NUT) carcinoma (formerly NUT midline carcinoma) (NC) is a poorly differentiated carcinoma, genetically defined by NUTM1 (NUT [nuclear protein in testis] midline carcinoma family member 1) gene rearrangement with fusion partners bromodomain-containing 4 (BRD4), bromodomain-containing protein 3 (BRD3), nuclear receptor binding SET domain protein 3 (NSD3), or other genes (NUT-variant carcinomas). NC most often arises in the thorax (mediastinum), head and neck. Although originally considered to be a neoplasm related to midline structures, it can involve a variety of anatomic sites and manifest in lateralized organs such as the lungs,^1^ kidney,^2^ and parotid gland,^3^ or in nonmidline locations.^4^ Locoregional and distant metastases are common, and more than 80% of the patients die within the first year of diagnosis.^5^ We illustrate 2 cases of NC, one displaying neuroendocrine features closely resembling a small cell lung cancer (SCLC), and the other one presenting with disseminated metastatic disease involving the bone marrow and mimicking hematological malignancy.

A 35-year old man presented with a rapidly growing lung tumor in the middle lobe enveloping the lateral segment bronchus. Transbronchial lung biopsies revealed a poorly differentiated carcinoma of small- to medium-sized cells arranged in solid sheets and with crush artifacts. The morphological appearance was reminiscent of SCLC (Figure 1A and B). Fine-needle aspirate (FNA) from the lung showed small- to medium-sized cells, dispersed in single cells and some in small clusters, with polygonal enlarged nuclei, granular chromatin, irregular nuclear contours, distinct nucleoli, and a moderate amount to sparse cytoplasm (Figure 1G). Crush artifacts and naked nuclei were identified, while keratinized cells were not seen. The tumor cells expressed synaptophysin (Figure 1C), cytokeratin MNF 116, cytokeratin 5/6 (CK5/6), cell adhesion molecule 5.2 (CAM5.2), p40, p63 (Figure 1E and F), without expression of thyroid transcription factor 1 (TTF-1), GATA binding protein 3 (GATA3), chromogranin A, CD56, CD45, or S100. The proliferation rate (Ki-67) was 25% to 35%. Programmed death ligand 1 (PDL1) (SP263, Ventana Assay) was expressed by 10% to 15% of the tumor cells. Immunohistochemistry for delta like canonical notch ligand 3 (DLL3) (SP347) was negative. Somatostatin receptor 2A was positive in 20%. NUT protein (monoclonal antibody, C52 Cell Signaling Technologies) revealed a specific diffuse nuclear speckled positivity (Figure 1D). Dual-color break-apart fluorescence in-situ hybridization (FISH) for NUTM1 performed on the cytological specimen confirmed the diagnosis of NC identifying rearrangements in 36 of 50 tumor cells (Figure 1H). He was still alive at the time of drafting the report and received chemotherapy.

**Figure 1.**
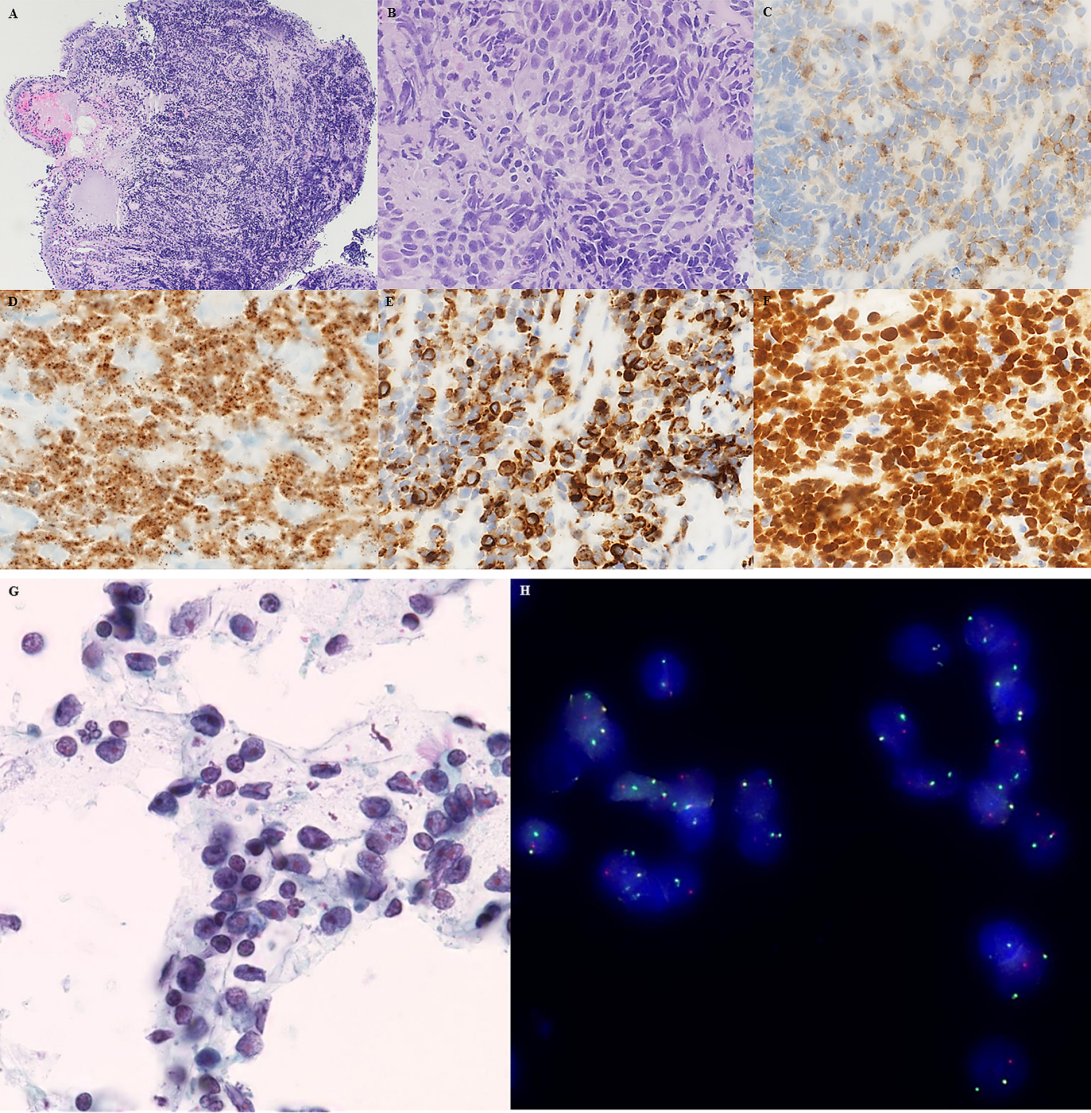
(A and B) Transbronchial lung biopsy showing infiltrates by primitive-appearing monotonous cells (hematoxylin and eosin) with nuclear molding, speckled chromatin, and crush artifacts. (C) Synaptophysin ( + ), (D) punctate NUT expression in the tumor cell nuclei ( + ), (E) expression of CK5/6 ( + ), and (F) uniform expression of p40 ( + ). (G) FNA specimen and (H) NUTM1 dual-color break-apart fluorescence in-situ hybridization showing fused signals and single orange/green signals in the majority of tumor cells.

Abbreviations: NUT, nuclear protein in testis; CK5/6, cytokeratin; FNA, fine-needle aspirate; NUTM1, NUT midline carcinoma family member 1.

A 72-year-old man presented with metastatic disease of unknown primary, bicytopenia (thrombopenia and anemia), and multiple tumor nodules in the lung, among them a larger mass-forming pulmonary nodule, a nodule in the right kidney, and extensive diffuse bone infiltration. Peripheral blood count and differential blood counts were as follows: leukocytes 22.27/mm^3^, thrombocytes 24/mm^3^, erythrocytes 3.73/mm^3^, hemoglobin 11.5 g/dL, eosinophils 0, basophils 1.0, monocytes 6.0, lymphocytes 18.0, myelocytes 2.5, metamyelocytes 2.5, and segmented neutrophils 37.0. Paraproteinemia was not detected. No enlarged lymph nodes, hepato-, or splenomegaly were noted on clinical inspection and imaging studies. Clinically, a hematological disease was suspected. The patient’s personal medical record included a history of autoimmune hepatitis treated with Imurek. A bone marrow biopsy 13 years earlier performed for evaluation of blood eosinophilia had shown normal trilinear hematopoiesis with increased eosinophils and reactive lymphoid infiltrates without evidence of lymphoma. In the current metastatic setting, a bone marrow biopsy from the left crista iliaca was performed and demonstrated an infiltrate by blastoid cells (Figure 2A and B), which were negative for myeloid and lymphoid markers (Figure 1C) and positive for CD99 (Figure 1D). The tumor cells were negative for BerEP4/EpCAM, pancytokeratin AE1/AE3 (Figure 1G), cytokeratin 19, cell adhesion molecule 5.2 (CAM5.2), epithelial membrane antigen (EMA), and friend leukemia virus integration 1 (FLI1). myogenic differentiation 1 (MYOD1), desmin, wilms tumor 1 (WT1), CD57, transducin-like enhancer of split 1 (TLE1), melanoma/prostate markers, chromogranin A, and inhibin were negative. SMARCB1/INI1 protein expression was retained. These results excluded rhabdomyo-, synovial, and epithelioid sarcoma. No sarcoma-specific translocation was identified using Archer^TM^FusionPlex^TM^Sarcoma Kit. Oncomine^TM^ Comprehensive Panel Version 3 revealed a nonpathogenic PMS2 p.G857A exon 15 and SETD2 p.T1033A exon 3 mutation. Testing for NUT protein (Figure 1E) (monoclonal antibody C52 Cell Signaling Technologies) and p63 (Figure 1F) was positive. Dual-color split apart FISH (Figure 1H) and Oncomine Focus Assay (TruSight RNA Pan Cancer Panel, Illumina) identified a BRD4-NUTM1 gene fusion resulting from t(15;19)(q14;p13) which confirmed the diagnosis of NC. The patient died during the evaluation process. The pulmonary nodules were not biopsied. The NC was assumed to originate from the lung with metastatic spread to the bone marrow and kidney.

**Figure 2.**
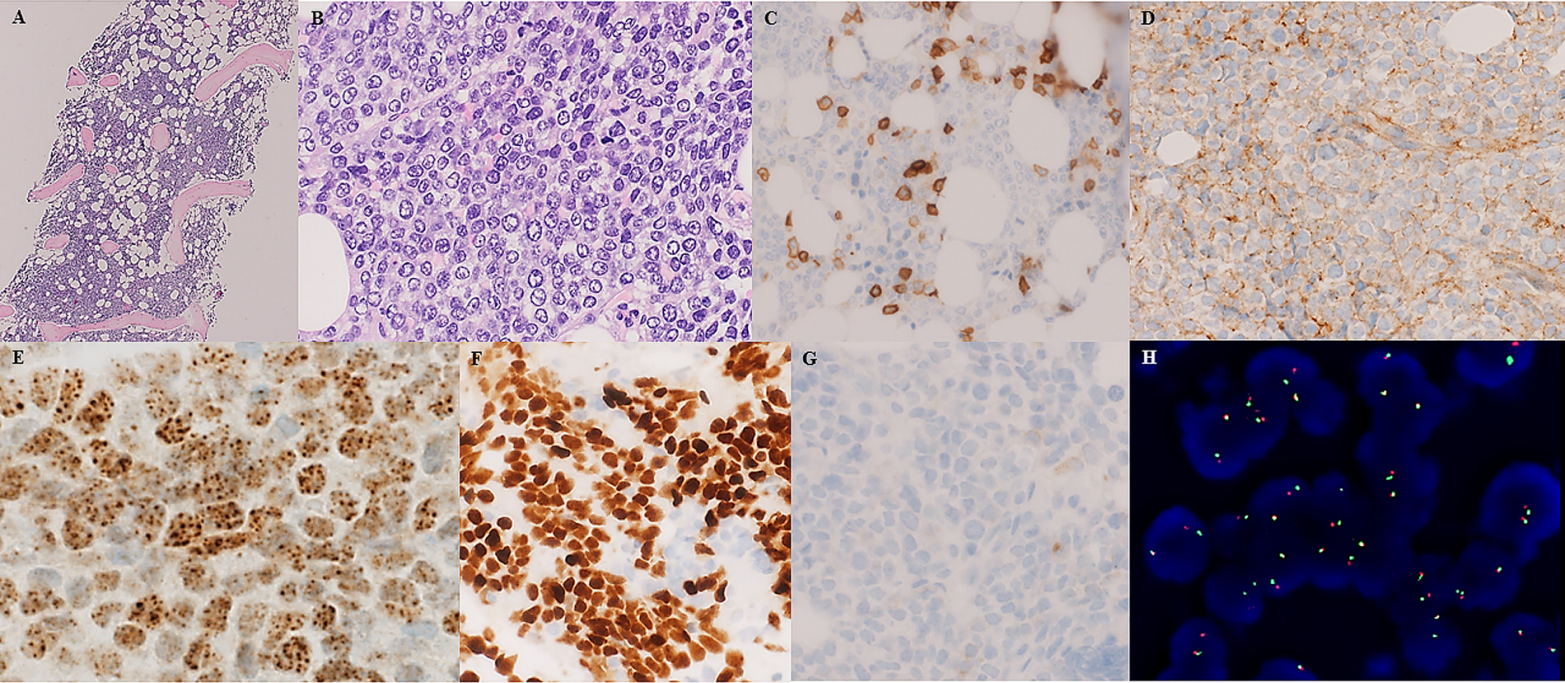
(A and B) Bone marrow biopsy showing diffuse infiltration by blastoid cells (hematoxylin and eosin). (C) CD45 ( − ) (scattered leukocytes positive), (D) CD99 ( + ), (E) NUT ( + ), (F) p63 ( + ), (G) pancytokeratin ( − ), and (H) NUTM1-FISH ( + ).

Abbreviations: NUT, nuclear protein in testis; NUTM1, NUT midline carcinoma family member 1; FISH, fluorescence in-situ hybridization.

NC are histologically characterized by sheets of undifferentiated cells with sparse eosinophilic or amphophilic cytoplasm, irregular nuclear contours, and prominent nucleoli. They are relatively uniform in size. Mitotic figures are brisk. Squamous differentiation is frequent and may appear as abrupt keratinization with sheets of immature cells juxtaposed to mature squamous nests.^6–9^ NC may share morphological similarities with neuroendocrine tumors. The nuclei may vary from pale open chromatin to hyperchromatic neuroendocrine-type appearance. Cell-to-cell molding and crush artifacts can be seen in NUT carcinoma, and it may express synaptophysin, focally CD56, and epithelial markers.^3^ The features of NC seen in histological specimens including nuclear size, shape, chromatin, nucleoli and cytoplasm are similar to the features seen in cytological specimens, although squamous cell differentiation is usually less obvious in cytological specimens compared to histological specimens. The cytological findings are characterized by high cellularity of the smears, medium-sized poorly to undifferentiated cells, arranged in single cells or patternless clusters, with round to oval nuclei and irregular nuclear contours. The cytoplasm is scant which makes the nuclei susceptible to nuclear molding and squeezing artifacts.

NC can manifest as diffuse bone infiltrate mimicking hematologic malignancy. It can express protein tyrosine phosphatase receptor type C (CD45RO) and CD34 and be confused with acute leukemia and other hematologic disorders.^10^ NC is often positive for EMA, p63, p40, pancytokeratin AE1/AE3, cytokeratin 5/6, but may be keratin-negative and CD99-positive mimicking sarcoma.^11,12^ Recent studies found that NUTM1 gene rearrangements can be found outside the classic clinicopathological spectrum of NC in myxoid spindle cell sarcoma or undifferentiated sarcoma, also with positive NUT immunoreactivity but with different fusion partners (myelin associated glycoprotein [MAG] and MAX dimerization protein 4 [MXD4]).^13^ Another study reported NUTM1 rearrangements in 6 primary undifferentiated tumors of soft tissue and viscera (brain, kidney, stomach wall)—2 of them with novel NUTM1 fusion partners (BCL6 corepressor like 1 [BCORL1] and MAX dimerization protein 1 [MXD1]).^14^ Despite overlaps with NC, the authors labeled these neoplasms NUT-associated tumors because of the differences in anatomic distribution, morphology, and immunophenotype when compared to NC. In contrast to NC, only 2 of the 6 cases had significant keratin and p63 expression, respectively. Both cases with novel NUTM1 fusion partners (BCORL1 and MXD1) did not show NUT immunoreactivity in this series.^14^ This observation is shared by other authors who found that NUT protein was inconsistently expressed by NUT-variant carcinomas, suggesting that variant fusion proteins may be expressed at lower levels than the BRD3 and BRD4 fusion products.^15^ Because NUT-variant carcinomas may not be susceptible to bromodomain inhibitor therapy, identifying the fusion partner may become necessary with respect to the prediction of responsiveness to bromodomain inhibitor therapy.^13^
